# Metformin: A Prospective Alternative for the Treatment of Chronic Pain

**DOI:** 10.3389/fphar.2020.558474

**Published:** 2020-09-23

**Authors:** Guadalupe Del Carmen Baeza-Flores, Crystell Guadalupe Guzmán-Priego, Leonor Ivonne Parra-Flores, Janet Murbartián, Jorge Elías Torres-López, Vinicio Granados-Soto

**Affiliations:** ^1^ Laboratorio de Mecanismos de Dolor, División Académica de Ciencias de la Salud, Universidad Juárez Autónoma de Tabasco, Villahermosa, Mexico; ^2^ Departamento de Farmacobiología, Cinvestav, South Campus, Mexico City, Mexico; ^3^ Departamento de Anestesiología, Hospital Regional de Alta Especialidad “Dr. Juan Graham Casasús”, Villahermosa, Mexico; ^4^ Neurobiology of Pain Laboratory, Departamento de Farmacobiología, Cinvestav, South Campus, Mexico City, Mexico

**Keywords:** anxiety, AMPK activation, chronic pain, metformin, depression, neuropathic pain, diabetic neuropathy, diabetes

## Abstract

Metformin (biguanide) is a drug widely used for the treatment of type 2 diabetes. This drug has been used for 60 years as a highly effective antihyperglycemic agent. The search for the mechanism of action of metformin has produced an enormous amount of research to explain its effects on gluconeogenesis, protein metabolism, fatty acid oxidation, oxidative stress, glucose uptake, autophagy and pain, among others. It was only up the end of the 1990s and beginning of this century that some of its mechanisms were revealed. Metformin induces its beneficial effects in diabetes through the activation of a master switch kinase named AMP-activated protein kinase (AMPK). Two upstream kinases account for the physiological activation of AMPK: liver kinase B1 and calcium/calmodulin-dependent protein kinase kinase 2. Once activated, AMPK inhibits the mechanistic target of rapamycin complex 1 (mTORC1), which in turn avoids the phosphorylation of p70 ribosomal protein S6 kinase 1 and phosphatidylinositol 3-kinase/protein kinase B signaling pathways and reduces cap-dependent translation initiation. Since metformin is a disease-modifying drug in type 2 diabetes, which reduces the mTORC1 signaling to induce its effects on neuronal plasticity, it was proposed that these mechanisms could also explain the antinociceptive effect of this drug in several models of chronic pain. These studies have highlighted the efficacy of this drug in chronic pain, such as that from neuropathy, insulin resistance, diabetic neuropathy, and fibromyalgia-type pain. Mounting evidence indicates that chronic pain may induce anxiety, depression and cognitive impairment in rodents and humans. Interestingly, metformin is able to reverse some of these consequences of pathological pain in rodents. The purpose of this review was to analyze the current evidence about the effects of metformin in chronic pain and three of its comorbidities (anxiety, depression and cognitive impairment).

## Introduction

Acute pain is considered an alarm system for protecting body integrity, while chronic pain may serve an adaptative role (Crook et al., 2014; Lister et al., 2020). In spite of its role, chronic pain is a major health problem around the world with a prevalence up to 50% (Fayaz et al., 2016; Manion et al., 2019). Chronic pain (defined in humans as the pain of more than three months) describes pain such as neuropathic pain, low-back pain, osteoarthritis, traumatic injury and postoperative pain, among others. Interestingly, chronic pain may induce anxiety and depression in rodents and humans (Wang et al., 2019; Zhou et al., 2019). In addition, chronic pain leads to cognitive impairment (Zhou et al., 2016; Shiers et al., 2018). Acute pain is mainly treated with non-steroidal anti-inflammatory drugs (NSAIDs) and/or opioids. In contrast, chronic pain is not easy to treat. Currently the pharmacotherapy for chronic pain includes tricyclic antidepressants (amitriptyline and nortriptyline), anticonvulsants (gabapentin and pregabalin) and opioids (tramadol and morphine). However, this pharmacotherapy has limited efficacy and several side effects. Although metformin was discovered in the 1940s, its properties as an anti-diabetic drug were known in 1957 (Sterne, 1957). There is evidence that metformin reduces weight, hyperglycemia and glycosylated hemoglobin in type 2 diabetic patients (Clarke and Duncan, 1968; Clarke and Campbell, 1977; Campbell and Howlett, 1995; DeFronzo and Goodman, 1995; Stumvoll et al., 1995; Johansen, 1999; Hundal et al., 2000) with a favorable risk-benefit assessment in patients with diabetes mellitus (Howlett and Bailey, 1999). In addition, a large multicenter study showed that metformin improves long-lasting metabolic effects and reduces cardiovascular risk (UKPDS, 1998). Later, several comprehensive reviews have been documented the effects of metformin in diabetes (Hermann, 1979; Vigneri and Goldfine, 1987; Dunn and Peters, 1995; Garber, 1997; Maruthur et al., 2016; Markowicz-Piasecka et al., 2017; Rena et al., 2017).

Recently, many pre-clinical studies have demonstrated the antinociceptive effects of metformin in rodents. Since this drug is a disease-modifying drug in type 2 diabetes, it is likely that it may share these properties to reduce pain and its comorbidities. Here, we assess the current state of evidence regarding the effects of metformin in chronic pathological pain and two of its comorbidities (anxiety/depression and cognitive impairment). Data strongly suggest that metformin could open a new avenue for the treatment of pathological pain and some of its associated comorbidities.

## Mechanisms of Action of Metformin

There is evidence that metformin decreases hepatic glucose production (Stumvoll et al., 1995; Hundal et al., 2000) and hepatic lipids (Lin et al., 2000), whereas it enhances myocyte glucose uptake (Hundal et al., 1992; Galuska et al., 1994). Although these effects of metformin have been known for many years, the underlying mechanisms remained unknown until 2001. AMP-activated protein kinase (AMPK) is a heterotrimeric serine/threonine enzyme composed of a catalytic α subunit and two regulatory (β, γ) subunits (Hardie et al., 1998; Kemp et al., 1999). There are two isoforms of the α and β subunits and three isoforms of the γ subunit, giving twelve possible combinations of the heterotrimeric αβγ AMPK complex. Currently, two upstream kinases account for the physiological activation of AMPK: liver kinase B1 (LKB1) and Ca^2+^/CaM-dependent protein kinase kinase 2 (CaMKK2). AMPK is activated by phosphorylation at threonine 172 in the α-catalytic subunit by LKB1 (Hawley et al., 2003; Woods et al., 2003; Shaw et al., 2004). In addition, AMPK is activated independently by direct binding with CaMKK2 (Green et al., 2011; Fogarty et al., 2016). Once phosphorylated/activated, AMPK phosphorylates and regulates key enzymes involved in metabolism and transcription factors that regulate gene expression. By that time, it was already known that AMPK activation led to stimulation of hepatic fatty acid oxidation and ketogenesis, inhibition of cholesterol synthesis, triglyceride synthesis, inhibition of adipocyte lipolysis and lipogenesis. Based on these actions, it was postulated in 1999 that activation of AMPK signaling could be the mechanism of action of metformin in the treatment of type 2 diabetes (Winder and Hardie, 1999). Then, the group of Zhou et al. reported for the first time a mechanism to explain the effects of metformin in diabetes. They discovered that metformin was able to induce its anti-diabetic effects by activation of AMPK in hepatocytes. This activation diminished the expression of lipogenic genes, promoted glucose uptake and suppressed SREBP-1 (insulin-stimulated transcription factor implicated in the pathogenesis of insulin resistance and type 2 diabetes) (Zhou et al., 2001). Metformin also inhibited mitochondrial respiratory chain complex I (Owen et al., 2000), which in turn guided to a reduction in cellular energy status and to up-regulation of the plasma-membrane glucose transporters (GLUT) 1 and 4 (Hundal et al., 1992; Fischer et al., 1995). This effect depended on the presence of LKB1 (Shaw et al., 2005). Others confirmed the effects of metformin on AMPK in humans (Musi et al., 2002). Later, other researchers found that AMPK activation inhibited in a mammalian target of rapamycin complex 1 (mTORC1)-dependent fashion phosphorylation of p70 ribosomal protein S6 kinase1 (p70S6K1) (Kimura et al., 2003) and phosphatidylinositol 3-kinase/protein kinase B (PI3-K)/Akt signaling pathway (Tzatsos and Kandror, 2006). Likewise, it was reported that metformin-mediated AMPK activation led to inhibition of mTORC1, p70S6K and 4E-BP1 and to reduction of cap-dependent translation initiation (Dowling et al., 2007). More recently, it was found that AMPK activation by metformin is abated in cells lacking inositol polyphosphate multikinase (IPMK), suggesting that LKB1 requires activation by IPMK to phosphorylate AMPK (Bang et al., 2014).

In contrast, other reports have described that metformin may induce its effects in an AMPK-independent manner (Gawler et al., 1989; Yu et al., 1994; Foretz et al., 2010; Miller et al., 2013). There is evidence that metformin may induce its effects by inhibiting the effects of glucagon, AMP-dependent protein kinase A (PKA) activity and phosphorylation of PKA substrates *in vitro* (Miller et al., 2013).

## Effects of Metformin on Pain

### Neuropathic Pain

Neuropathic pain is caused by a lesion or disease of the somatosensory nervous system (Jensen et al., 2011). This pain results from traumatic nerve, spinal cord, or brain injury, as well as diabetes, human immunodeficiency virus, and post-herpetic viral infection or with multiple sclerosis, cancer or chemotherapeutic drugs. Neuropathic pain is notoriously resistant to the actions of NSAIDs and opioids, whereas other drugs have only partial effects (Finnerup et al., 2010; Finnerup et al., 2015).

Previous studies have demonstrated that mTORC1 is a regulator of protein synthesis. Of note, mTORC1 signaling can be inhibited by rapamycin, thus preventing downstream signaling (Hay and Sonenberg, 2004; Takei et al., 2004; Zhang et al., 2014). By controlling protein translation, mTORC1 regulate the activity of sensory neurons in the periphery and central nervous system (Asante et al., 2009; Géranton et al., 2009). Reinforcing this, mTORC1 and their downstream targets of the translational machinery are localized in a subset of A-fiber nociceptors (Klann and Dever, 2004; Piper and Holt, 2004; Raab-Graham et al., 2006; Jiménez-Díaz et al., 2008; Obara et al., 2015). Accordingly, the mTORC1 inhibitor rapamycin reduces capsaicin-, nerve injury- or spinal cord injury-induced hypersensitivity in rodents (Jiménez-Díaz et al., 2008; Codeluppi et al., 2009; Géranton et al., 2009). Moreover, intrathecal administration of rapamycin reduces formalin-induced pain-related behavior (Kim et al., 1998; Price et al., 2007; Asante et al., 2009). These data suggest that the mTORC1 pathway plays a key role in the translation and protein synthesis in primary afferent neurons which maintains chronic plasticity in pathological pain. With these antecedents, the group of Price and co-workers was the first to demonstrate the antinociceptive effect of metformin in pain (Melemedjian et al., 2011). They found that treatment with metformin starting 2- or 7-weeks post-nerve injury completely reversed spared nerve injury (SNI)-induced tactile allodynia in mice. Moreover, metformin diminished spinal nerve ligation (SNL)-induced tactile allodynia in rats (Melemedjian et al., 2011). Authors of this study reported that metformin was able to activate AMPK and inhibited the mTORC1 pathway in mouse trigeminal (TG) neurons. Likewise, metformin reversed nerve growth factor (NGF)-induced hyperexcitability of mouse TG neurons in culture. In addition, metformin inhibited eukaryotic translation initiation factor 4F (eIF4F) formation in primary cultures of TG and dorsal root ganglia (DRG) neurons treated with NGF. These data demonstrate that metformin suppresses nerve injury-induced aberrant translation pathways in primary afferent neurons, reduces neuronal excitability and inhibits pain in models of neuropathic pain in mice and rats (Melemedjian et al., 2011). In support of this, other studies from the same group reported that daily injection with metformin in mice with SNI 7-weeks post-injury reverses tactile allodynia by diminishing mTORC1 and extracellular regulated protein kinase (ERK) activation pathways in sensory neurons. This antiallodynic effect of metformin persisted for at least 2 months (Melemedjian et al., 2013a). Since ERK activation in the peripheral nervous system is a well-known mechanism for increasing the excitability of nociceptors (Ji et al., 2009), the observed effects of metformin may be due, in part, to the inhibition of ERK-induced phosphorylation of Nav1.7 sodium channels (Stamboulian et al., 2010). Interestingly, repeated administration of metformin reduces tactile allodynia and increases apolipoprotein E expression, which is linked to functional recovery after nerve injury (Melemedjian et al., 2013a; Melemedjian et al., 2013b). Furthermore, metformin was able to prevent other neuropathic pain types in rodent models such as cisplatin-, paclitaxel-and bortezomib-induced tactile allodynia (Mao-Ying et al., 2014; Wei et al., 2017; Falcão-Pereira et al., 2019; Ludman and Melemedjian, 2019) or spinal cord injury (Zhang et al., 2017; Liu et al., 2019; see **Table 1**). Some of these effects are mediated by reducing elevated cytokines levels (interleukin-1β and TNFα), suppressing the expression of the signal transducer and activator of transcription 3 (p−STAT3) and inhibiting activation of microglia and astrocytes at the spinal cord (Afshari et al., 2018; Ge et al., 2018). Other authors have demonstrated that metformin diminishes neuropathic pain, enhances autophagy markers (LC3 and beclin1) and promotes accumulation of autophagy substrate protein p62 in the ipsilateral spinal cord (Weng et al., 2019). The Price’s group found recently that AMPK activation inhibits nascent protein synthesis and increases P-body formation (RNA granules) in DRG neurons. They also reported that neuropathic pain decreases P-bodies in the DRG, consistent with an increased mRNA translation, in mice, whereas that metformin restores these effects in neuropathic animals (Paige et al., 2019). These data add to the evidence that metformin induces its antiallodynic effects by reducing the aberrant translation induced by nerve injury. Of note, the effect of metformin in neuropathic pain is sexually dimorphic (Inyang et al., 2019). Although metformin is able to activate AMPK at the same extent in neurons and microglia of male and female rats, this drug does not affect nerve injury-induced neuropathic pain in female rats. Authors suggest that this divergence could be due to the OCT2 expression between males and females (Inyang et al., 2019).

**Table 1:** Summary of studies about the antinociceptive effect of metformin in models of neuropathic, inflammatory and dysfunctional pain in rodents.

Pain model	Effects of metformin	Mechanisms of metformin	Species	Doses and administration route (References)
**Spared nerve injury (SNI, mice) and spinal nerve ligation (SNL, rats)**	Reversed mechanical allodynia induced by nerve injury	AMPK activation. Inhibited mTOR pathway and the eIF4F complex formation in TG neurons. Reduced nascent protein synthesis in sciatic nerve	Male ICR and C57BL/6 mice Male Sprague-Dawley rats	200 mg/kg/day for 7 days, ip (Melemedjian et al., 2011)
**Inflammatory pain (formalin test and zymosan A test)**	Prevented development of inflammatory pain	AMPKα2 subunit activation	Male C57BL/6 mice	100 mg/kg, ip (Russe et al., 2013)
**Spared nerve injury, SNI (mice)** **Spinal nerve ligation, SNL (rats)**	Diminished tactile allodynia	AMPK activation. Prevented rapamycin-induced ERK activation and suppressed mTOR/p70S6 kinase signaling in sensory neurons	Male ICR and C57BL/6 mice Male Sprague-Dawley rats	200 mg/kg/day for 7 days, ip(Melemedjian et al., 2013a)
**Nerve injury (SNI, mice and SNL, rats)**	Reduced tactile allodynia	Increased Apolipoprotein E expression in sciatic nerve	Male ICR mice Male Sprague-Dawley rats	200 mg/kg/day for 7 days, ip(Melemedjian et al., 2013b)
**Chemotherapy-induced peripheral neuropathy by cisplatin and paclitaxel**	Prevented the development of mechanical allodynia	Reduced loss of peripheral nerve endings of intra-epidermal nerve fibers	C57Bl/6J mice	200 mg/kg/day for 7 days, ip(Mao-Ying et al., 2014)
**Diabetic neuropathy induced by streptozotocin (STZ)**	Reduced thermal hyperalgesia and tactile allodynia	No determined	Male Wistar rats	500 mg/kg/day for 4–6 weeks, po (Yadav et al., 2014)
**Diabetic neuropathy induced by STZ**	Reduced diabetes-induced mechanical hyperalgesia, heat hyperalgesia and cold allodynia	Decreased malondialdehyde and glycation end products levels in blood. Increased superoxide dismutase activity and expression of p-AMPK, PGC-1α, Sirt-3, and nNOS in sciatic nerves	Male Sprague-Dawley rats	30–500 mg/kg, ip (Ma et al., 2015)
**High fat diet/STZ (HFD/STZ) insulin resistance**	Prevented mechanical hyperalgesia	No determined	Male Sprague-Dawley rats	200 mg/kg/day for 5 weeks, po (Byrne et al., 2015)
**Chronic inflammatory pain induced by compound C**	Reduced thermal hyperalgesia induced by compound C	Increased p-AMPK, restored serum levels of IL-1β and IL-18	Male C57/BL6/J mice	100 ng/kg/day for 1 week, ip (Bullón et al., 2016)
**STZ-and methylglyoxal-induced pain**	Inhibited methylglyoxal-induced nociception and STZ-induced tactile allodynia	No determined	Male Wistar rat	250 mg/kg, sc (Huang et al., 2016)
**Post-surgical pain (plantar incision)**	Inhibited incision-evoked mechanical hypersensitivity and hyperalgesic priming induced by PEG_2_	AMPK activation in DRG neurons in culture	Male ICR mice	30–200 mg/kg/day for 4 days, ip (Burton et al., 2017)
**Chemotherapy-induced neuropathy (bortezomib)**	Reduced bortezomib-induced tactile allodynia	Prevented the increase of RAGE induced by bortezomib in spinal dorsal horn	Male Sprague-Dawley rats. Male C57 mice	25–50 µg/10 µl/day, for 10 days, it (Wei et al., 2017)
**Spinal cord injury (SCI)**	Improvement of functional locomotor activity after SCI	Activated AMPK and inhibited mTOR signaling. Improved functional recovery through autophagy flux stimulation	Female Sprague-Dawley rats	50 mg/kg/day, ip (Zhang et al., 2017)
**Chronic constriction injury (CCI)**	Chronic treatment reversed established thermal hyperalgesia	Activated AMPK and suppressed p-STAT3 expression Inhibited Iba-1 and GFAP expression induced by CCI in spinal dorsal horn	Male Sprague-Dawley rats	200 mg/kg/day for 10 days, ip (Ge et al., 2018)
**Spared-nerve injury (SNI)**	Reversed the pain-related cognitive impairment in male mice	Restored axon initial segment in infralimbic cortex	Male and Female C57BL/6J mice	200 mg/kg/day for 7 days, ip Shiers et al., 2018
**Spinal cord injury (SCI)**	Decreased sensitivity to mechanical and thermal allodynia induced by SCI	Attenuated TNFα and IL-1β levels in spinal cord	Male Sprague-Dawley rats	10–100 mg/kg, ip (Afshari et al., 2018)
**Type2 diabetes neuropathy**	Reduced tactile allodynia	Decreased number of synapses in the L5 segment of spinal dorsal horn	Male adult Sprague-Dawley rats	200 mg/kg/day for 28 days, po (Lin et al., 2018)
**Type2 diabetes neuropathy induced by neonatal STZ**	Alleviated tactile allodynia	No determined	Male Wistar rats	200 mg/kg/day for 2 weeks, po (Barragán-Iglesias et al., 2018)
**Methylglyoxal-induced pain.** **Type1 diabetes induced by STZ**	Reduced tactile allodynia	AMPK activation. Reduced eIF2α^Ser51^ phosphorylation in IB4+ DRG neurons	Male eIF4E^S209A^ and C57BL/6 mice. Male Wistar rats	200 mg/kg/day for 10 days, po (Barragán-Iglesias et al., 2019)
**Spared nerve injury (SNI)**	Prevented and reverts SNI-induced mechanical and cold hypersensitivity	Decreased microglial activation in dorsal horn from male but not female mice	Male and female mice	200 mg/kg/day for 7 days, ip (Inyang et al., 2019)
**Nociceptive response and chronic constriction injury (CCI)**	Increased latency to hot-plate test. Attenuated mechanical allodynia	Naltrexone partially attenuated the antinociceptive effect of metformin	Female Swiss mice	500 or 1000 mg/kg, po (Augusto et al., 2019)
**Nerve injury (SNL)**	Attenuated SNL‐induced mechanical and thermal hyperalgesia	Prevented SNL‐induced apoptosis. Enhanced autophagy markers LC3 and beclin1 in dorsal horn	Male Sprague‐Dawley rats	5 mg/kg/day, ip (Weng et al., 2019)
**Nerve injury (SNI)**	Inhibited nascent protein synthesis and increased processing-body formation in DRG obtained from SNI mice	Increased levels of Rck/p54 containing RNA granules in DRG in culture	Male ICR mice	200 mg/kg, ip (Paige et al., 2019)
**Neuropathy induced by chemotherapy (bortezomib)**	Prevented development of bortezomib-induced mechanical allodynia	Reduced levels of hypoxia-inducible factor 1 alpha (HIF1A) in DRG limiting the translation of hypoxia-inducible factor 1α (HIF1A)	Male ICR mice	150 mg/kg, ip (Ludman and Melemedjian, 2019)
**Neuropathic pain by fructose-induced insulin resistance**	Acute and chronic treatment reduced tactile allodynia	Chronic treatment reversed fructose-induced changes in Insulin receptor β, ASIC3, anoctamin-1, and ATF3 expression	Male Wistar rats	50–200 mg/kg/day, for 4 weeks, po (García et al., 2019)
**Radiculopathy by lumbar disc herniation (LDH)**	Alleviated LDH-induced pain hypersensitive behaviors	AMPK activation. Suppressed mTOR/p70S6K signaling in DRG neurons	Male Wistar rats	250 mg/kg/day, ip (Liu et al., 2019)
**Visceral Allodynia induced by LPS**	Reduced visceral allodynia	AMPK activation, nitric oxide, and central D2 receptors	Male Sprague-Dawley rats	5–50 mg/kg/day for 3 days, ip (Nozu et al., 2019)
**Pain-related hypersensitivity to heat and mechanical stimuli. Capsaicin and formalin test**	Attenuated pain-related hypersensitivity in Cntnap2^−/−^ mice	Restored the hyperactive Akt-mTOR signaling in DRG neurons from Cntnap2^−/−^ mice	Contactin-associated protein-like 2 knock-down mice (Cntnap2^−/−^)	200 mg/kg/day for 2 days, ip (Xing et al., 2020)
**Complex regional pain syndrome fracture model**	Reduced mechanical allodynia	No determined	C57BL/6J female mice	200 mg/kg/day for 7 days, ip Das et al., 2020

mTOR. mammalian target of rapamycin; eIF4F, eukaryotic translation initiation factor 4F; PGC-1α, peroxisome proliferator-activated receptor gamma coactivator-1α; Sirt-3, Sirtuin 3; nNOS, neuronal nitric oxide synthase; ERK, extracellular regulated protein kinase; IL-1β, interleukin-1β; p-STAT3, signal transducers and activators of transcription 3; Iba-1, ionized calcium binding adapter 1; GFAP, glial fibrillary acid protein; LC3, autophagy-related protein; Rck/p54, P body marker and translational repressor/decapping activator; ASIC3, acid-sensing ion channel 3; ATF3, activating transcription factor 3.

### Insulin Resistance

Pre-diabetes represents the earliest stage of glucose dysregulation and precedes the development of overt type 2 diabetes. Several studies have found that nerve injury-induced neuropathic pain develops before the establishment of high blood glucose levels (hyperglycemia) (Ziegler et al., 2008; Ziegler et al., 2009; Papanas and Ziegler, 2012; Lee et al., 2015). Metabolic syndrome is the aggregation of dyslipidemia, reduced high‐density lipoprotein cholesterol, central obesity, insulin resistance (pre-diabetes or diabetes) and hypertension (Stino and Smith, 2017). Mounting evidence demonstrates that high-fat diet can serve as experimental model of obesity, increased fat mass, and insulin resistance (Guilford et al., 2011; Groover et al., 2013; Lupachyk et al., 2013; Cooper et al., 2016; Cooper et al., 2018). High-fat diet increases body weight, fat deposition, mildly increases blood glucose and induces hyperinsulinemia and tactile allodynia (Groover et al., 2013; Lupachyk et al., 2013; Cooper et al., 2016; Cooper et al., 2018; Prakash et al., 2020). Accordingly, metformin counteracts these effects by AMPK activation and transforming growth factor-β1 signaling inhibition in white adipose tissue of rodents and humans (Luo et al., 2016). Interestingly, regular exercise promotes weight reduction and antinociception by activation of AMPK (King-Himmelreich et al., 2017; Slivicki et al., 2019). Likewise, caloric restriction reduces tactile allodynia by stimulating AMPK-mediated autophagy (Coccurello et al., 2018), whereas ketogenic diet induces accumulation of energy by products such as AMP and ADP that in turn activate AMPK (Xiao et al., 2007). All these interventions have a common mechanism of action, activation of AMPK. Several studies suggest that AMPK activation reduces eukaryotic translation initiation factor 2 subunit α (eIF2α) phosphorylation in several cell types (Dong et al., 2010; Liang et al., 2013; Boß et al., 2016). Since eIF2α regulates ternary complex availability (eIF4F complex) (Trinh and Klann, 2013), it has been suggested that metformin also reduces eIF2α-dependent translation initiation (Price and Géranton, 2009; Khoutorsky and Price, 2018; Uttam et al., 2018; Megat and Price, 2018). A recent study from our laboratory shows that metformin diminishes chronic fructose-induced tactile allodynia (a model of insulin resistance). Moreover, the same study demonstrates that metformin reduces ATF3 (nerve injury marker), anoctamin-1 and acid-sensing ion channels 3 (ASIC3), whereas it restores insulin receptor-β, α_5_ subunit containing GABA_A_ receptors (α_5_GABA_A_ receptors) receptors and tandem pore domain acid-sensitive K^+^ channel 3 (TASK-3) protein expression in DRG and sciatic nerve (García et al., 2019). Whether metformin induces these effects by activating AMPK is not known.

### Diabetic Neuropathy

Type 2 diabetes is recognized as a global epidemic with an incidence that continues to rise. Diabetic neuropathy is the most common cause of neuropathy worldwide. This pathology affects approximately half of patients with diabetes and it increases with age. The painful symptoms of diabetic neuropathy are commonly severe and often lead to depression, anxiety, sleep disorders and reduced quality of life (Pop-Busui et al., 2017). To date, there is not an effective disease-modifying pharmacotherapy to treat the condition. However, several preclinical studies suggest that metformin or other AMPK activators could be used for the treatment of diabetic neuropathy. For instance, it has been shown that metformin diminishes diabetes-induced mechanical hyperalgesia, heat hyperalgesia and cold allodynia and restores streptozotocin-induced changes in hyperglycemia, weight loss, glucose intolerance, reduction of nerve conduction velocity, malondialdehyde, glycation end-products levels, glycosylated hemoglobin levels and superoxide dismutase activity (Yadav et al., 2014; Byrne et al., 2015; Ma et al., 2015; Hasanvand et al., 2016; Barragán-Iglesias et al., 2018; García et al., 2019). Since hyperglycemia leads to formation of methylglyoxal (Bierhaus et al., 2012; Jack et al., 2012) and oxidative stress, it is likely that streptozotocin induces nociception in rats by activating the integrated stress response (ISR) trough reducing the activity of AMPK (Barragán-Iglesias et al., 2019). Accordingly, systemic administration of metformin inhibits paw-injected methylglyoxal-induced nociception, blocks streptozotocin-induced tactile allodynia (Huang et al., 2016; Barragán-Iglesias et al., 2019) and activates AMPK (Melemedjian et al., 2011; Ma et al., 2015). It has been proposed that hyperglycemia-derived methylglyoxal depends on activation of Nav1.8 sodium and transient receptor potential ankyrin 1 (TRPA1) channels (Bierhaus et al., 2012; Huang et al., 2016). Authors suggest that methylglyoxal could also activate the RAGE/STAT3 signaling pathway in dorsal horn, which in turn participates in central sensitization and persistent pain (Wei et al., 2017). These effects, however, are reached at high methylglyoxal concentrations. Our group has found that low concentrations of methylglyoxal stimulate the ISR and increases eIF2α^Ser51^ phosphorylation in IB4+ nociceptive neurons of the DRG of mice *in vivo* and *in vitro*. In support of this, the specific inhibitor (ISRIB) of the ISR diminishes eIF2α^Ser51^ phosphorylation and reduces and reverts methylglyoxal-induced nociception and also reduces type 1 diabetes-induced neuropathic pain in mice and rats. Interestingly, the AMPK activator drug metformin also lessens eIF2α^Ser51^ phosphorylation (Barragán-Iglesias et al., 2019). These data imply that metformin can decrease the effects of methylglyoxal on pain by reducing eIF2α^Ser51^ phosphorylation in an AMPK-dependent manner (see **Table 1**). Taken together, data suggest that metformin behaves as a disease-modifying drug in insulin resistance- and experimental diabetes-induced pathological pain.

### Inflammatory Pain

Although acute inflammatory pain is essential for protecting our bodies from potential damage, chronic inflammatory pain, which lasts for 3 months or longer, serves an adaptative role but still represents a pathological disease. Up to 50% of patients who undergo surgical procedures develop chronic pain (Johansen et al., 2012). It is believed that neuronal plasticity consists of peripheral sensitization in DRG neurons and TG and central sensitization of neurons in the spinal cord and brain (Basbaum et al., 2009; Luo et al., 2014). These processes are promoted by descending facilitatory pathways and central neuroinflammation, which mediate the persistence and chronicity of pain conditions (Ji et al., 2013).

Inhibition of spinal mTOR with rapamycin or metformin reduces carrageenan-, compound C (AMPK inhibitor)- or complete Freund’s adjuvant (CFA)-induced hyperalgesia and allodynia and restores all changes (up-regulation of mTORC1, p70S6K, 4E-BP1, NF-κβ and cytokines) induced by activation of the mTOR signaling (Norsted-Gregory et al., 2010; Bullón et al., 2016; Xiang et al., 2019). These data suggest that the mTORC1 signaling also participates in inflammatory pain, while AMPK acting upstream reduces pain and carrageenan-induced long-lasting neuronal plasticity. Other studies have found that systemic administration of metformin prevents surgical incision-induced tactile allodynia and PGE_2_ injection-induced development of hyperalgesic priming in mice by activating AMPK (Burton et al., 2017). Thus, metformin is effective in reducing the transition to a chronic pain state. Interestingly, other AMPK activators, like resveratrol (Dasgupta and Milbrandt, 2007), also reduce tactile allodynia and inhibit hyperalgesic priming. These data add to a body of evidence that AMPK activation reduces development of acute tactile allodynia resulting from tissue injury (Tillu et al., 2012; Russe et al., 2013; Bullón et al., 2016), diminishes the excitability of nociceptors (Melemedjian et al., 2011; Tillu et al., 2012) and prevents the development of hyperalgesic priming (Mejia et al., 2016, see **Table 1**). Reinforcing this, metformin is not able to induce antinociception in AMPKα_2_ KO mice (Russe et al., 2013). Interestingly, the antinociceptive effect of metformin is similar to that of exercise. Exercise leads to the increase of anandamide that in turn activates AMPK to induce antinociception (King-Himmelreich et al., 2017). This mechanism may also explain the effect of exercise in several types of pain (Slivicki et al., 2019).

### Pain in Humans

There are only a few studies assessing the effect of metformin in human beings. One retrospective study found that treatment with metformin in 46 diabetic patients with lumbar radiculopathy pain show lower levels of radiculopathy pain (Taylor et al., 2013). There is evidence to support that alterations in AMPK in fibroblasts from fibromyalgia patients could play an important role in this pathology. These alterations include diminished AMPK phosphorylation, decreased mitochondrial biogenesis, reduced oxygen consumption, decreased antioxidant enzymes expression levels and mitochondrial dysfunction. In these conditions, metformin is able to decrease mitochondrial dysfunction in fibroblasts from fibromyalgia patients *via* activation of AMPK (Alcocer-Gomez et al., 2015). In support of this, there are low ATP levels and high mitochondrial reactive oxygen species in bone marrow cells, as well as high levels of IL-1β and IL-18 in serum of fibromyalgia patients. Interestingly, all these biochemical alterations were restored to control values when bone marrow cells from patients with fibromyalgia are treated with metformin. Furthermore, this drug improves clinical symptoms (pain, fatigue, depression, disturbed sleep, and tender points) in these patients (Bullón et al., 2016). There is a need to explore the effects of metformin in patients with several types of chronic pain in order to validate the findings of preclinical studies.

### Anxiety and Depression

Mounting evidence suggests the existence of a positive correlation between insulin resistance or diabetes and anxiety and depression (Rasgon and Kenna, 2005; Sharma and Fulton, 2013; Zemdegs et al., 2016; Zemdegs et al., 2019). For instance, the induction of insulin resistance or experimental diabetes in rodents through exposure to high-fat diet leads to symptoms of depression including anxiety, despair and anhedonia (Gariepy et al., 2010; Ho et al., 2012; André et al., 2014). Several studies also suggest that neuropathic pain is associated with stress, anxiety and depression (Yalcin et al., 2011; Descalzi et al., 2017; Hestehave et al., 2019; Humo et al., 2019; Murasawa et al., 2020) whereas that chronic stress exacerbates chronic pain (Narita et al., 2006; Li et al., 2017; Gruener et al., 2018; Sieberg et al., 2018). These comorbidities have considerably increased contributing to disability, sleep disturbances, poor quality of life and healthcare costs. Since metformin can reverse several experimental insulin resistance- or diabetes-, and neuropathic pain-induced changes, it is likely that this drug may diminish anxiety-like behavior in rodents and humans. Regarding insulin resistance and diabetes, metformin induces a rapid anxiolytic effect, activates AMPK, up-regulates FoxO3a protein and GABA_A_ receptors expression and increases miniature inhibitory postsynaptic currents (Fan et al., 2018; Ji et al., 2019). Metformin also increases serotonin release in the ventral hippocampus, activity of serotonergic neurons in the dorsal raphe nucleus and promotes anxiolytic/antidepressant-like activities of mice fed a high-fat diet (Zemdegs et al., 2019). Likewise, metformin diminishes chronic stress-induced depression-like behaviors in mice (Fang et al., 2020). Authors suggest that this effect could be due to the activation of AMPK.

Regarding neuropathic pain, there is no studies showing the effect of metformin in nerve injury-induced anxiety/depression. Treatment with mirogabalin (3–10 mg/kg) relieves nerve injury-related anxiety-type behaviors and tactile allodynia (Murasawa et al., 2020), suggesting that reduction of neuropathic pain could lead to a reduction of the comorbidities. Pre-clinical studies have suggested that forced physical exercise decreases neuropathic pain in rodents (Kuphal et al., 2007; Chen et al., 2012; Groover et al., 2013; Guo et al., 2019). Exercise improves physical function and fibromyalgia symptoms (Schachter et al., 2003). As stated above, there are some studies suggesting that physical exercise reduces weight and pain by activation of AMPK in mice with neuropathic pain (King-Himmelreich et al., 2017; Slivicki et al., 2019). Since exercise reduces nociception in neuropathic rodents and humans, it is likely that this non-pharmacological intervention could also decrease nerve injury-induced anxiety- and depression-like behaviors in neuropathic patients. Considering that half of chronic pain patients have comorbid anxiety and depression (McWilliams et al., 2004), there is a necessity to develop pharmacological treatments which can reduce chronic pain and comorbidities. Thus, the possible therapeutic use of metformin could open a new avenue for the treatment of these comorbidities (Anderson et al., 2010; Erensoy et al., 2019). Whether metformin reduces comorbidities by reducing pain or other mechanisms is currently unknown.

### Cognitive Deficits

There has been reported a link between neuropathic pain (including diabetic neuropathy) and cognitive deficits in rodents (Kodama et al., 2011; Shiers et al., 2018; Boccella et al., 2019; Liang et al., 2020; Won et al., 2020) and humans (Curatolo et al., 2017; Ojeda et al., 2018; Naranjo et al., 2019). However, current therapies for neuropathic pain have not considered this relationship. Recent pre-clinical studies show that cisplatin or paclitaxel induces neuropathic pain and deficits in spatial orientation and memory (cognitive impairment) in rodents and these effects are prevented by metformin. Moreover, this drug prevents tactile allodynia and restores chemotherapy-induced changes in white matter organization, neuronal arborization, and dendritic spine density (Zhou et al., 2016). In this sense, metformin abates pain-related cognitive impairment and restores functional and morphological changes in brain of neuropathic mice. In contrast, gabapentin is able to decrease pain, but not cognitive impairment (Shiers et al., 2018). Likewise, chronic treatment with metformin reduces nociception and cognitive dysfunction in high-fat diet-induced insulin resistance. This drug also decreases all metabolic changes induced by a high-fat diet in rodents (Pintana et al., 2012; Lennox et al., 2014; Muñoz-Arenas et al., 2020; Zhang et al., 2020). Interestingly, chronic treatment with metformin for 24 weeks improves cognitive performance, reduces depression and metabolic changes induces by type 2 diabetes in human beings (Guo et al., 2014; Ng et al., 2014). More basic and clinical studies are necessary to fully elucidate if metformin is effective to reduce chronic pain and cognitive deficits in neuropathic pain.

## Pharmacokinetics of Metformin

Metformin gastrointestinal absorption is apparently complete within 6 hours of ingestion (Scheen and Paquot, 2012). It is absorbed in the small intestine, particularly in jejunum and ileum. The intestinal absorption of metformin is mediated by the plasma membrane monoamine transporter (PMAT) expressed on the luminal side of enterocytes and organic cation transporter (OCT) 3 (OCT3, *SLC22A3*) expressed on the brush border of the enterocytes (Müller et al., 2005; Zhou et al., 2007; Graham et al., 2011). Once in the blood, the drug reaches the liver primarily by OCT1 (*SLC22A1*) and OCT3 (*SLC22A3*) expressed on the basolateral membrane of hepatocytes (Nies et al., 2009; Chen et al., 2010; Graham et al., 2011) in order to induce its anti-diabetic effects (Zhou et al., 2001; Rena et al., 2017). Pharmacokinetic-pharmacodynamic modeling has shown a correlation between the time course of metformin concentrations in the portal vein and gut wall and hypoglycemic effect, instead of drug concentrations in liver (Stepensky et al., 2002; Sun et al., 2011). The time to reach maximal plasma concentrations in human beings after metformin administration (T_max_) is 1.5 to 2.7 h (Caillé et al., 1993; Sambol et al., 1996; Wei et al., 2009; Zhang et al., 2014; McCreight et al., 2018), which follows a multiphasic pattern (Graham et al., 2011), giving a peak plasma concentration (C_max_) of 1.1 to 2.5 µg/ml (Wei et al., 2009; Zhang et al., 2014; Dias et al., 2019), and a steady-state concentration range of 0.3 to 1.5 µg/ml (see **Table 2**). Plasma protein binding is negligible, and the drug is not metabolized (Scheen, 1996).

**Table 2:** Pharmacokinetics of metformin in humans, horses and rats.

Species	C_max_ (µg/ml)	T_max_ (h)	t_1/2_ (h)	Vd (L)	Cl (ml/min)	Bioavailability (%)		Reference
						IV	PO	
Human	ND	ND	1.5 ± 0.3	62.7 ± 7.7	440.8 ± 89	*		Sirtori et al., 1978
Human	1.5 ± 0.2	1.9 ± 0.4	2.6 ± 0.1	ND	444.0 ± 23.0		51.6	Pentikäinen et al., 1979
Human	3.2 ± 0.9	2.1 ± 0.8	5.2 ± 0.6	ND	322.0 ± 166.0		50-60	Tucker et al., 1981
Human	0.6 ± 0.1	2.4 ± 0.9	3.16 ± 0.4	ND	ND		ND	Caillé et al., 1993
Human	1.8 ± 0.2	2.6 ± 0.2	4.6 ± 0.7	367 ± 41.6	968.3 ± 91.7		12	Sambol et al., 1996
Horses	ND	ND	24.9 ± 0.4	141.3 ± 6^€^	5720 ± 524	*		Hustace et al., 2009
Human	1.9	1.6	2.0	148.3 ± 5.1^£^	50.0 ± 0.08^¥^		ND	Wei et al., 2009
Human	3.4 ± 0.6	2.3 ± 0.6	3.5 ± 1.5	ND	ND		ND	Homsek et al., 2010
Human	2.4 ± 1.0	1.9 ± 1.0	3.6 ± 1.0	ND	ND		ND	Chen et al., 2011
Human	1.0	4.0	ND	ND	ND		ND	Hussey et al., 2013
Human	1.1 ± 0.2	1.5 ± 1.0	3.5 ± 0.6	ND	967 ± 316		ND	Kim et al., 2014
Rats	2.5 ± 0.1	1.0	7.2 ± 0.5	ND	ND		ND	Elango et al., 2015
Rats	ND	ND	4.8 ± 2.5	2.3 ± 1.4^€^	29.5 ± 6.0^§^	*		Gabr et al., 2017
Rats	1.4 ± 0.1	2	3.4 ± 0.3	ND	ND		ND	Paul et al., 2017
Human	2.1	ND	4.8	197.3	28.6 ^ϕ^		95	McCreight et al., 2018
Human	1.9 ± 0.8	1.5 ± 1.0	3.1 ± 1.5	ND	430.3 ± 113.1		ND	Chung et al., 2018
Human	1.1 ± 0.5	3.5 ± 0.7	6.0 ± 0.6	ND	ND		ND	Mohamed et al., 2019
Rats	7.4 ± 0.8	1.0	2.8 ± 1.4	ND	ND		ND	Wu et al., 2019
Rats	2.3 ± 0.3	2.0 ± 0.4	2.8 ± 1.2	1.7 ± 0.3	0.93 ± 0.26^ϕ^		ND	Elgawish et al., 2019
Rats	17.0 ± 5.7	2.3 ± 0.6	4.2 ± 1.1	ND	32 ± 11^§^		ND	Lyu et al., 2019
Human	3.2 ± 1.8	4.0 ± 0.6	ND	ND	ND		ND	Nikolaidis et al., 2020

Data are the mean ± standard error. ND, Not determined.

^€^L/kg, ^£^µl, ^¥^ml/h, ^§^ml/min/kg, ^ϕ^L/h, *100%.

Once in blood, metformin enters the kidney cells through OCT1 and OCT2 expressed in the basolateral membrane (Kim et al., 2014). Metformin undergoes rapid and biphasic renal excretion (Melchior and Jaber, 1996). Renal excretion of this drug from the tubule cell to the lumen is mediated through multidrug and toxin extrusion protein 1 (MATE1, *SLC47A1*) and 2K (MATE2K, *SLC47A2*) expressed on the apical membrane of the renal proximal tubule cells (Sato et al., 2008; Tsuda et al., 2009; Ito et al., 2012). The elimination half-life (t_1/2_) is between 1.5 and 7 h (Tucker et al., 1981; Caillé et al., 1993), or longer if renal function is impaired (Caillé et al., 1993; Dias et al., 2019). Metformin is eliminated by glomerular filtration and tubular secretion. According to the clinical trials, the renal clearance of metformin is around 50 ml/h (Wei et al., 2009). Plasma concentrations of metformin decrease rapidly after intravenous administration (Graham et al., 2011). The clearance (Cl/F) range from 441 to 706 ml/min in healthy volunteers (Sirtori et al., 1978; Pentikäinen et al., 1979; Tucker et al., 1981; see **Table 2**).

Pharmacokinetic parameters can be modified after two different secondary doses of metformin (250 or 1000 mg). In this case, C_max_ varied from 591.1 ± 247.5 to 1937.5 ± 863.0 µg/ml), whereas T_max_ and t_1/2_ did not show dose-dependent changes (Chung et al., 2018). A recent study showed that high-intensity interval exercise diminishes metformin concentration (2–3 h), increases C_max_ (4.4 ± 2.5 µg/ml) and diminishes T_max_ (2.7 ± 0.9 h) (Nikolaidis et al., 2020). Moreover, metformin pharmacokinetics is altered by severe renal impairment (Scheen, 1996), metformin intolerance (McCreight et al., 2018), but not by diabetes (Sambol et al., 1996; Markowicz-Piasecka et al., 2017; see **Table 2**).

## Side Effects of Metformin

There is evidence that metformin treatment in human beings leads to gastrointestinal side effects including diarrhea, dyspepsia and flatulence in up to 30% of patients (Melchior and Jaber, 1996; Florez et al., 2010; Ji et al., 2015; Tanaka et al., 2015). Diarrhea has also been reported in rats with a dose of 200 to 250 mg/kg/day during 5 weeks (Acosta-Cota et al., 2019; Takemori et al., 2020). However, it not usual that pain studies report side effects. Thus, this is an important point to consider in future studies of pain.

## Synopsis

**Figure 1:**
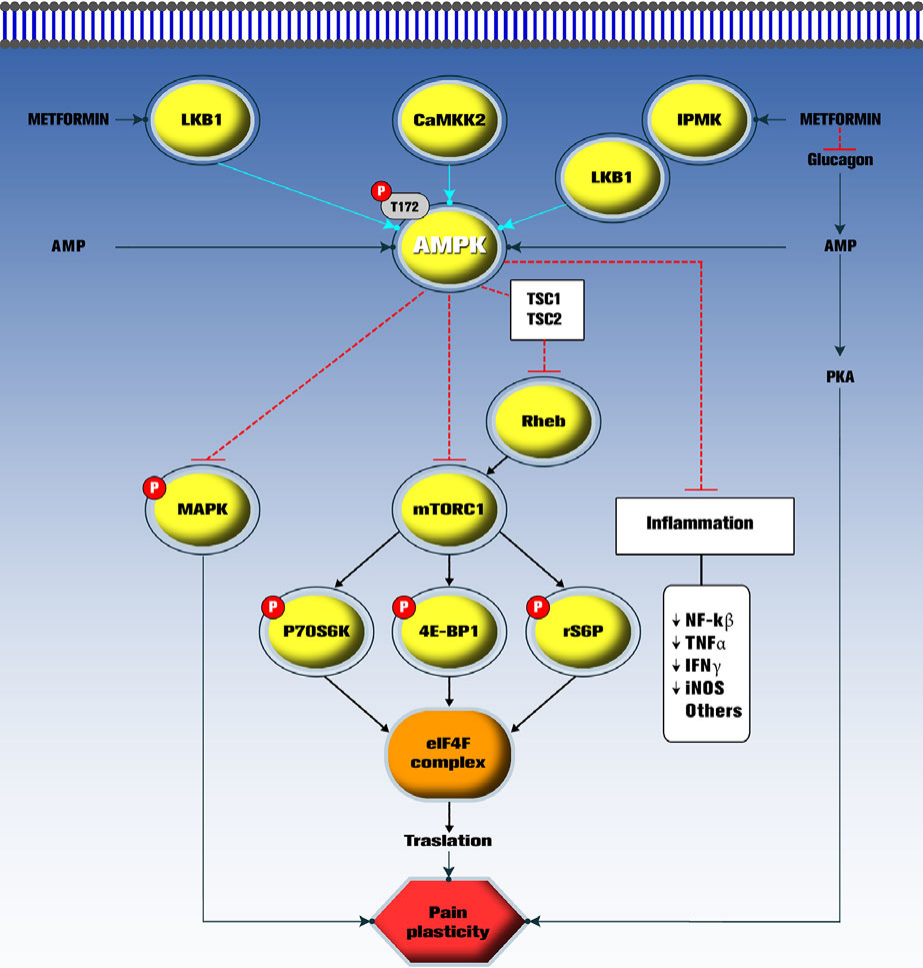
Current mechanisms proposed for metformin in pathological pain. The mTOR/P70S6K/4E-BP1/rS6P pathway activates formation of the eIF4F complex and promotes aberrant translation in nociceptors during pathological pain. Also, activation of mitogen-activated protein kinase (MAPK), inflammation and protein kinase A (PKA) pathways lead to pain plasticity. Metformin acts as a disease-modifying drug by indirectly activating AMPK. Once activated, phosphorylated AMPK inhibits the mTORC1 pathways reversing pain plasticity and pathological pain. Metformin also inhibits glucagon actions to induce its antihyperglycemic effect in diabetic conditions. However, the role of this pathway on pain has not been explored. LKB1, Liver kinase B1; CaMKK2, Calcium/calmodulin-dependent protein kinase kinase 2; IPMK, Inositol polyphosphate multikinase; AMPK, AMP-activated protein kinase; Rheb, GTP-bound Rheb GTPase; mTORC1, Mechanistic target of rapamycin complex 1; P70S6K, 70 kDa ribosomal protein S6 kinase; 4E-BP1, Eukaryotic translation initiation factor 4E (eIF4E)–binding protein 1; rS6P, S6 ribosomal protein; eIF4F: Eukaryotic translation initiation factor 4F; nNOS, Neuronal nitric oxide synthase; NF-kβ: Nuclear factor kappa β; TNFα, Tumor necrosis factor α; IFN*γ*, interferon-*γ*.

We have summarized the main pharmacological characteristics of metformin, a highly used drug in diabetic persons. This drug has been useful to treat people with pre-diabetes, metabolic syndrome and diabetes. Comprehensive data on pain indicate that metformin behave as a disease-modifying drug, as it targets a master switch kinase (AMPK) which in turn decreases the activity of mTORC1 and MAPK signaling in nociceptors and reduces pain in several models of pathological pain (Price and Dussor, 2013; Price et al., 2016; Asiedu et al., 2017; **Figure 1**). Moreover, metformin has pharmacological effects in rodent models of anxiety, depression and cognitive impairment. Interestingly, the mechanism of action of metformin has been unraveled in the last years. There is evidence to support that AMPK activation signaling underlies the effects of this drug in several pathologies including insulin resistance, diabetes and chronic neuropathic pain (**Figure 1**), although the role of AMPK in pain-related anxiety/depression and cognitive impairment is unknown. Also, it is unknown if metformin directly decreases comorbidities or it decreases pain and this reduction halts comorbidities. Of note, well-designed placebo-controlled clinical trials are needed to support the putative effect of metformin in preclinical studies, particularly in neuropathic pain and its comorbidities.

## Author Contributions

All authors contributed to the article and approved the submitted version.

## Funding

This work was partially supported by Conacyt (grant CB-2017-2018/A1-S-40015 to VG-S).

## Conflict of Interest

The authors declare that the research was conducted in the absence of any commercial or financial relationships that could be construed as a potential conflict of interest.

## Abbreviations

α_5_GABA_A_ receptor, α_5_ subunit containing GABA_A_ receptor; 4E-BP1, eukaryotic translation initiation factor 4E binding protein 1; AMPK, AMP-activated protein kinase; ASIC3, acid-sensing ion channels 3; ATF3, Activating factor transcription 3; CaMKK2, Calcium/calmodulin-dependent protein kinase kinase 2; CFA, Complete Freund’s adjuvant; C_max_, peak plasma concentration; DRG, dorsal root ganglia; eIF2α, eukaryotic translation initiation factor 2 subunit α; eIF2α^Ser51^, eukaryotic translation initiation factor 2α phosphorylated in serine 51; eIF4F, eukaryotic translation initiation factor 4F; ERK, extracellular regulated protein kinase; GLUT1, glucose transporter 1; GLUT4, glucose transporter 4; IL-18, Interleukin-18; IL-1β, interleukin-1β; IPMK, Inositol polyphosphate kinase; ISR, integrated stress response; LKB1, Liver kinase B1; MATE1, multidrug and toxin extrusion protein 1; MATE2K, multidrug and toxin extrusion protein 2K; mTORC1, mammalian target of rapamycin complex 1; NF-κB, nuclear factor kappa B subunit; NGF, nerve growth factor; OCT1, organic cation transporter 1; OCT3, organic cation transporter 3; p70S6K1, p70 ribosomal protein S6 kinase1; PGE_2_, Prostaglandin E_2_; PI3-K/Akt, phosphatidylinositol 3-kinase/protein kinase B; PKA, AMP-dependent protein kinase A; PMAT, plasma membrane monoamine transporter; p−STAT3, phosphorylated signal transducer and activators of transcription 3; SCI, spinal cord injury; SNI, spared nerve injury; SNL, spinal nerve ligation; STAT3, signal transducer and activator of transcription 3; t_1/2_, Half-life; TASK-3, tandem pore domain acid-sensitive K^+^ channel 3; TG, trigeminal ganglia; T_max_, time to reach the maximal concentration; TRPA1, transient receptor potential ankyrin 1.
